# High miR-30 Expression Associates with Improved Breast Cancer Patient Survival and Treatment Outcome

**DOI:** 10.3390/cancers13122907

**Published:** 2021-06-10

**Authors:** Maral Jamshidi, Rainer Fagerholm, Taru A. Muranen, Sippy Kaur, Swapnil Potdar, Sofia Khan, Eliisa Netti, John-Patrick Mpindi, Bhagwan Yadav, Johanna I. Kiiski, Kristiina Aittomäki, Päivi Heikkilä, Jani Saarela, Ralf Bützow, Carl Blomqvist, Heli Nevanlinna

**Affiliations:** 1 Department of Obstetrics and Gynecology, University of Helsinki and Helsinki University Hospital, 00029 Helsinki, Finland; maral.jamshidi@helsinki.fi (M.J.); rainer.fagerholm@helsinki.fi (R.F.); taru.a.muranen@helsinki.fi (T.A.M.); sippy.kaur@helsinki.fi (S.K.); sofia.khan@iki.fi (S.K.); eliisa.netti@helsinki.fi (E.N.); johanna.kiiski@helsinki.fi (J.I.K.); ralf.butzow@hus.fi (R.B.); 2 Department of Pathology, University of Helsinki and Helsinki University Hospital, 00029 Helsinki, Finland; paivi.heikkila@hus.fi; 3 Institute for Molecular Medicine Finland (FIMM), University of Helsinki, 00014 Helsinki, Finland; swapnil.potdar@helsinki.fi (S.P.); john.mpindi@siemens-healthineers.com (J.-P.M.); bhagwan.yadav@helsinki.fi (B.Y.); jani.saarela@helsinki.fi (J.S.); 4 Turku Bioscience Centre, University of Turku and Åbo Akademi University, 20520 Turku, Finland; 5 Department of Clinical Genetics, University of Helsinki and Helsinki University Hospital, 00029 Helsinki, Finland; kristiina.aittomaki@helsinki.fi; 6 Department of Clinical Genetics, HUS Diagnostic Center, HUSLAB, 00029 Helsinki, Finland; 7 Department of Pathology, HUS Diagnostic Center, HUSLAB, 00029 Helsinki, Finland; 8 Department of Oncology, University of Helsinki and Helsinki University Hospital, 00029 Helsinki, Finland; carl.blomqvist@helsinki.fi

**Keywords:** breast cancer, metastasis, survival, miR-30, HER-2, p53, chemotherapy, doxorubicin, anthracycline, lapatinib

## Abstract

**Simple Summary:**

Previous research on the miR-30 family and breast cancer patient survival and on miR-30-related chemosensitivity prompted us to design a comprehensive study on the role of the miR-30 family in general and on miR-30d in particular in breast cancer. We present a study consisting of a tumor microarray analysis of 1238 breast cancer patients, a survival analysis, a drug-sensitivity screen with six breast cancer cell lines, and an in-silico pathway analysis. In our analysis, high miR-30d expression was associated with improved survival in breast cancer patients with aggressive tumor phenotypes. In the drug-sensitivity analysis, ectopic expression of miR-30 family members sensitized the cell lines to the treatment. The pathway analysis based on miRNA and mRNA expression in the METABRIC data suggested that the miR-30 family may have an inhibitory role in pathways contributing to EMT and metastasis. Our results suggest prognostic and predictive potential for the miR-30 family for further investigation.

**Abstract:**

Deregulated miRNA expression has been suggested in several stages of breast cancer pathogenesis. We have studied the miR-30 family, in particular miR-30d, in relation to breast cancer patient survival and treatment outcomes. With tumor specimens from 1238 breast cancer patients, we analyzed the association of miR-30d expression with tumor characteristics with the 5-year occurrence of breast cancer-specific death or distant metastasis (BDDM), and with 10-year breast cancer survival (BCS). We conducted a two-stage drug-screen to investigate the impact of miR-30 family members (miR-30a-30e) on sensitivity to doxorubicin and lapatinib in six breast cancer cell lines HCC1937, HCC1954, MDA-MB-361, MCF7, MDA-MB-436 and CAL-120, using drug sensitivity scores (DSS) to compare the miR-30 family mimics to their specific inhibitors. The study was complemented with Ingenuity Pathway Analysis (IPA) with the METABRIC data. We found that while high miR-30d expression is typical for aggressive tumors, it predicts better metastasis-free (*p*_BDDM_ = 0.035, HR = 0.63, 95% CI = 0.4–0.9) and breast cancer-specific survival (*p*_BCS_ = 0.018, HR = 0.61, 95% CI = 0.4–0.9), especially in HER2-positive (*p*_BDDM_ = 0.0009), ER-negative (*p*_BDDM_ = 0.003), p53-positive (*p*_BDDM_ = 0.011), and highly proliferating (*p*_BDDM_ = 0.0004) subgroups, and after adjuvant chemotherapy (*p*_BDDM_ = 0.035). MiR-30d predicted survival independently of standard prognostic markers (*p*_BDDM_ = 0.0004). In the drug-screening test, the miR-30 family sensitized the HER2-positive HCC1954 cell line to lapatinib (*p* < 10^−2^) and HCC1937, MDA-MB-361, MDA-MB-436 and CAL120 to doxorubicin (*p* < 10^−4^) with an opposite impact on MCF7. According to the pathway analysis, the miR-30 family has a suppressive effect on cell motility and metastasis in breast cancer. Our results suggest prognostic and predictive potential for the miR-30 family, which warrants further investigation.

## 1. Introduction

Despite advances in early detection and treatment, breast cancer remains the leading cause of cancer death among women worldwide [1]. Therefore, it is crucial to identify robust predictive markers of survival and treatment outcome. Given that miRNAs (microRNAs) are often located at genomic regions involved in tumorigenesis [2], their expression analysis presents an attractive approach to identifying novel prognostic and drug response predictors [3,4]. A single miRNA can repress the expression of several gene products, albeit only to a modest degree [5,6], and thus, might be involved in a variety of cellular pathways. The deregulation of miRNAs disturbs gene expression and potentially promotes carcinogenesis [7]. Altered miRNA expression may contribute to the initiation, progression, and metastasis of breast cancer [8,9]. Several miRNAs have been associated with the pathogenesis of breast cancer, and based on the mechanism of their action, they can be classified as oncomiRs [10,11], tumor-suppressors [12], metastamiRs (pro-metastatic) [13], or metastasis-suppressors [14].

A miRNA array profiling study of breast (*n* = 73) and ovarian (*n* = 109) tumors by Li et al. identified 55 and 166 miRNAs, respectively, with DNA copy number amplification in more than 15% of the cases [11]. Of the 41 miRNAs amplified in both breast and ovarian tumors, they selected miR-30d (located at 8q21) as a candidate miRNA which was advantageous for cell growth in a long-term culture of an ovarian cancer cell line. Their clinical study suggested that higher expression of miR-30d predicts poor clinical outcomes in ovarian cancer patients [11]. Based on miRBase [15], the miR-30 family includes miR-30a-30e with extremely high sequence homology and 100% conservation in the seed region. Therefore, the rest of the family members, too, might be interesting candidates for prognostic and drug response studies.

We first applied in situ hybridization to assess miR-30d expression in a series of unselected and familial invasive breast cancer tumors to study its association with the clinical and pathological characteristics of the tumors and patient survival. We used drug sensitivity screening to explore the effect of the miR-30 family members on drug response in breast cancer cell lines in vitro, and a pathway enrichment analysis to identify the potential underlying molecular mechanisms.

## 2. Materials and Methods

### 2.1. Patients

MiR-30d expression was analyzed in tumors from 884 unselected breast cancer patients and 542 additional familial cases. The unselected cases were ascertained at the Department of Oncology, Helsinki University Hospital, during the years 1997–1998 and 2000 as previously described [16,17]. The additional familial breast cancer cases were collected by a systematic screening at the Department of Oncology, Helsinki University Hospital or were ascertained through genetic counseling at the Department of Clinical Genetics. The study cohort included 1238 invasive breast carcinomas available for TMA as previously described [18]. BRCA1 and BRCA2 mutations had been excluded from the familial patient series as described [19,20,21]. Clinical and pathological information on tumor characteristics, size, nodal status, and distant metastasis, as well as estrogen and progesterone receptor status were collected from the pathology reports. A breast cancer pathologist (P.H.) re-reviewed all tumors for histological diagnosis and grade using the Scarff–Bloom–Richardson method, as modified by Elston and Ellis [22]. HER2 status was obtained from TMAs using Chromogenic In Situ Hybridization (CISH) (0–1 = neg, 2–3 = pos); if CISH results were not available, immunohistochemistry (IHC) was used (0–1 = neg, 3 = pos; 2 = not used) [23]. The breast cancer tissue microarray sections were immunohistochemically stained for p53 expression using a mouse monoclonal anti-human p53-antibody (DO-7, DAKO) as described [18]. Samples were considered positive when >20% of the cancer cells were stained. The expression of Ki-67 was evaluated using a Ki-67 antibody [24]; samples were considered negative and positive when <20% and >20% of the cancer cells were stained, respectively. Information on adjuvant treatment and distant metastases during the follow-up was collected from the patient records. The information on death due to breast cancer or other reasons was obtained from the Finnish Cancer Registry. A total number of 659 tumors were of incident cases (entering the study less than 6 months from the date of diagnosis), which were included in the survival analysis.

### 2.2. miRNA In Situ Hybridization

In situ detection of miRNA expression was done on formalin-fixed paraffin-embedded tissue microarray sections as previously described [11] using the double digoxigenin labeled mir-30d locked nucleic acid probe (5′-CTTCCAGTCGGGGATGTTTACA-3′, 2.5 μM; Exiqon) in hybridization solution. The evaluation of the results was performed without knowledge of the clinico-pathological information. No staining, weak, moderate and strong cytoplasmic signals were recorded accordingly. These four categories were combined into two analysis groups (“low expression” = absent and low signal; “high expression” = intermediate and high signal), because there was no difference in the clinico-pathological associations or in the survival rates between tumors with absent or low signal, nor between tumors with intermediate or high signal.

### 2.3. Gene Expression and Pathway Enrichment Analyses

We utilized the processed METABRIC dataset (*n* = 1142) available as EGAS00000000122 in the European Genome-phenome archive [25]. The mRNA expression data (Illumina HT-12 v3 platform) was normalized using quantile normalization against single target distribution, as described [26]. The co-expression of the miR-30 family miRNAs and all curated mRNA-probes were analyzed using Spearman’s rank correlation, so that mRNA-probes with absolute correlation coefficient >0.25 were included in the functional enrichment analyses using the IPA (Ingenuity Pathway Analysis QIAGEN Inc., Aarhus, Denmark). We performed two parallel analyses: First, we focused on the genes negatively correlated with the miR-30 family members in the METABRIC data (Spearman rank correlation < −0.25). With the IPA *microRNA Target Filter*, we filtered the gene list to include only the algorithmically predicted or experimentally validated targets of each miR-30 family member and performed a functional enrichment analysis. Second, we identified all mRNAs co-expressed with the miR-30 family miRNAs (absolute value of Spearman rank correlation > 0.25) in the METABRIC data. The correlated genes were fed into the *IPA core analysis* for functional enrichment, using the Spearman correlation coefficients as surrogates for expression log ratio.

### 2.4. Sensitization Screening and Drug Response

The cell lines were confirmed for identity by the Genomics Unit of Technology Centre, Institute for Molecular Medicine Finland (FIMM), Helsinki, Finland, with the Promega StemElite^TM^ ID System (Promega Corp., Madison, Wi, USA) and further tested for the mycoplasma-free status. Assay-ready cells from MCF-7, MDA-MB-361, CAL-120, MDA-MB-436, HCC1937, and HCC1954 were prepared. Other potential cell lines, e.g., T47D, ZR-75-1, and BT474, were not successfully transfected during the preliminary transfectability-test. The cell line preparation was performed by culturing the cells in a large batch and aliquoting them into ampules kept in liquid nitrogen in solution containing 90% FBS and 10% DMSO. Immediately prior to transfection, the cells were thawed and washed with culture medium, and the cell number was counted using a hemocytometer. The cells were dissolved in medium into the required density for transfection. We performed a miRNA mimic versus-inhibitor-based drug response analysis in breast cancer cell lines. MiRNA mimics are double-stranded oligonucleotides, which mimic the corresponding miRNA. MiRNA inhibitors are single-stranded oligonucleotides that bind and inactivate their target miRNA, irreversibly. A custom human miRNA library was acquired from Ambion (mirVana™ miRNA mimic/inhibitor) (Table S1), and screened with 6 replicates in the first round and 11 replicates in the second round using 384-well plates. The intra-plate controls used in the screens were from Qiagen and Ambion, namely, pre-miR negative control#2 (32 replicates), and All-Stars-Death positive control (12 replicates), per 384-well plate. The final concentration of miRNA in assay plates was 10 nM. Doxorucibin (Sigma-Aldrich Solutions, Merck, Darmstadt, Germany) was added to transfected cells 24 h after transfection. The doxorubicin concentrations were 1, 10, 100, 500, 1000, and 10,000 nM and lapatinib was used at 0.83, 10, 100, 1000, 5000, and 10,000 nM concentration. Based on cell viability at increasing drug concentrations, drug response curves were calculated and converted into drug sensitivity score (DSS) statistics [27,28], which were pooled, and the miRNAs mimics’ and inhibitors’ results were compared using Student’s *t*-test.

### 2.5. Analysis of Tumor Phenotype and Patient Survival

Statistical analyses were conducted in R version 3.6.1 (R core team, Vienna, Austria). The significance limit was set at 0.05 (two-sided test). *p*-Values for evaluation of proportional differences in miR-30d expression by tumor characteristics were calculated using Pearson’s chi-squared test, or using Fisher´s exact test when *n* < 5 in any category of cross-tabulated tumor histology and miR-30d expression. Log-rank tests were used to assess the statistical significance of differences between Kaplan–Meier curves for survival analyses among patients. The patient survival was monitored until a breast cancer-associated death (BD-), or the occurrence of a distant metastasis (-DM). Patients lost from follow-up or alive 5 years after the diagnosis were right-censored, resulting in a measurement of 5-year BDDM. In the 5-year BDDM, 26 patients who had metastasis at the time of diagnosis were not included in the analyses. For 10-year breast cancer survival (BCS), the follow-up time was measured between the date of diagnosis and the date of death due to breast cancer and right-censored at 10 years. Univariate Cox regression analysis was used to estimate survival hazard ratios (HR) overall and in subgroups. Multivariate Cox regression was used to evaluate the independence of miR-30d in relation to common prognostic factors. Multivariate model covariates included grade (1,2,3—linear), tumor size, nodal status (negative/positive—dichotomous), M status (metastasis at diagnosis), estrogen receptor (negative/positive—dichotomous), progesterone receptor (negative/positive—dichotomous), and Ki-67 status (negative/positive—dichotomous). For 5-year BDDM, we did not include the M status in the multivariate model.

## 3. Results

Altogether, 1238 invasive breast carcinomas were available for tissue microarray (TMA) and in situ hybridization as described [18]. MiR-30d in situ hybridization results were obtained for 1193 (96.3%) tumors. 361 (30.3%) tumors showed no or low-intensity and 832 (69.7%) tumors showed high-intensity cytoplasmic staining (Figure S1). The remaining 45 (3.7%) of tumors were not scored due to loss of cores in the process or cores not containing enough tumor material. Figure S2 illustrates the flow chart of samples through the analyses.

### 3.1. High miR-30d Expression Is Associated with Aggressive Clinical and Pathological Features of the Tumors

High miR-30d expression was associated with tumors of ductal histopathological type (*p* = 0.003), high grade (*p* = 0.0002), positive nodal status (*p* = 0.007) and high proliferation rate as estimated by Ki-67 (*p* = 0.014) compared to tumors with low miR-30d expression. In hormone receptor-positive tumors (ER+ and/or PgR+), high miR-30d expression was associated with HER2 positive status (HER2+) (*p* = 0.032) (Table 1).

### 3.2. High miR-30d Expression Is an Independent Predictor of Improved Survival in Breast Cancer Patients and among Subgroups

**Figure 1:**
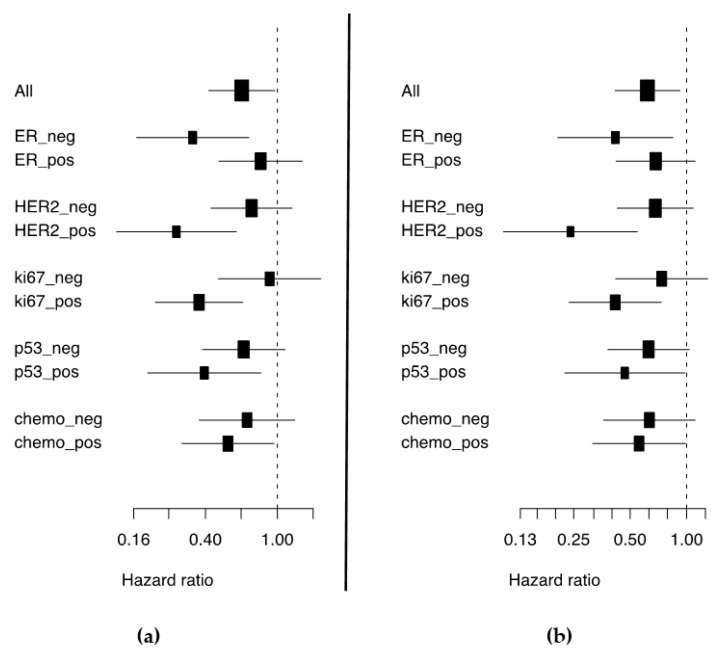
Forest plots depicting the hazard ratio and corresponding confidence intervals for (**a**) 5-year BDDM and (**b**) 10-year BCS by high expression of miR-30d.

**Figure 2:**
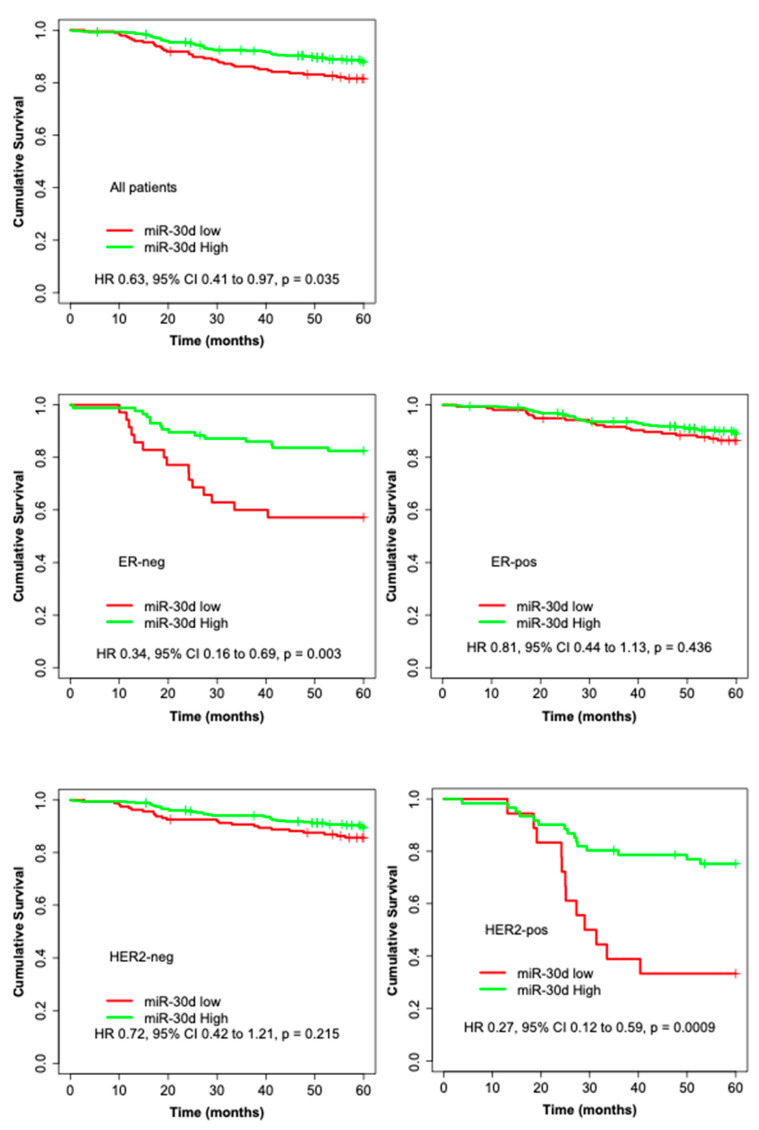

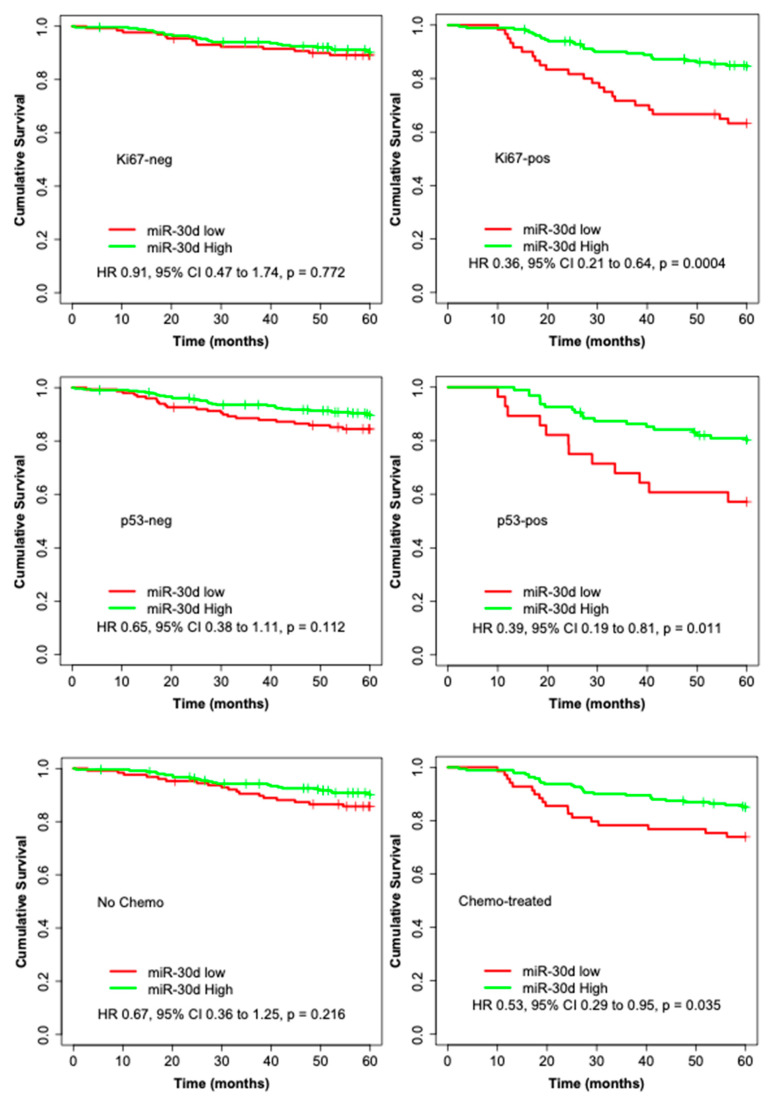
Kaplan–Meier curves illustrating the 5-year BDDM survival analyses by high and low expression of miR-30d for all patients and among the subgroups of: ER-negative, ER-positive, HER2-negative, HER2-positive, Ki67-negative, Ki67-positive, p53-negative, p53-positive, no chemotherapy, and chemotherapy-treated.

Only the incident breast cancer cases were included in the survival analyses, to avoid the over-representation of long-term survivors. Of the 659 cases, 205 (31.1%) were categorized into the low expression group and 454 (68.9%) into the high expression group. Table S2 shows the clinico-pathological features of the tumors of incident cases and their association with miR-30d expression. High miR-30d expression was associated with better 5-year metastasis-free survival (BDDM) (HR 0.63, 95% CI 0.41 to 0.97, *p*_BDDM_ = 0.035) and improved 10-year breast cancer survival (BCS) (HR 0.61, 95% CI 0.41 to 0.92, *p*_BCS_ = 0.018). High miR-30d expression was also associated with better survival among subgroups of patients with ER-negative tumors (*p*_BDDM_ = 0.003, *p*_BCS_ = 0.015), HER2-positive tumors (*p*_BDDM_ = 0.0009, *p*_BCS_ = 0.0006), highly proliferating tumors indicated by high expression of Ki-67 (*p*_BDDM_ = 0.0004, *p*_BCS_ = 0.002), p53-positive tumors (*p*_BDDM_ = 0.011, *p*_BCS_ = 0.043), and chemotherapy-treated patients (*p*_BDDM_ = 0.035, *p*_BCS_ = 0.045) (Figure 1 and Figure 2, Table S3). In multivariate Cox regression models adjusted for established prognostic markers (grade, tumor size, nodal status, and ER) as well as for factors significant in univariate analyses (HER2, Ki-67, and p53), the miR-30d expression remained an independent prognostic factor for 5-year BDDM and 10-year BCS (HR 0.46, 95% CI 0.30 to 0.70, *p*_BDDM_ = 0.0002; BCS, HR 0.54, 95% CI 0.35 to 0.84, *p*_BCS_ = 0.001)(Table 2).

### 3.3. miR-30 Family Members Sensitized Breast Cancer Cell Lines to Doxorubicin and Lapatinib

**Figure 3:**
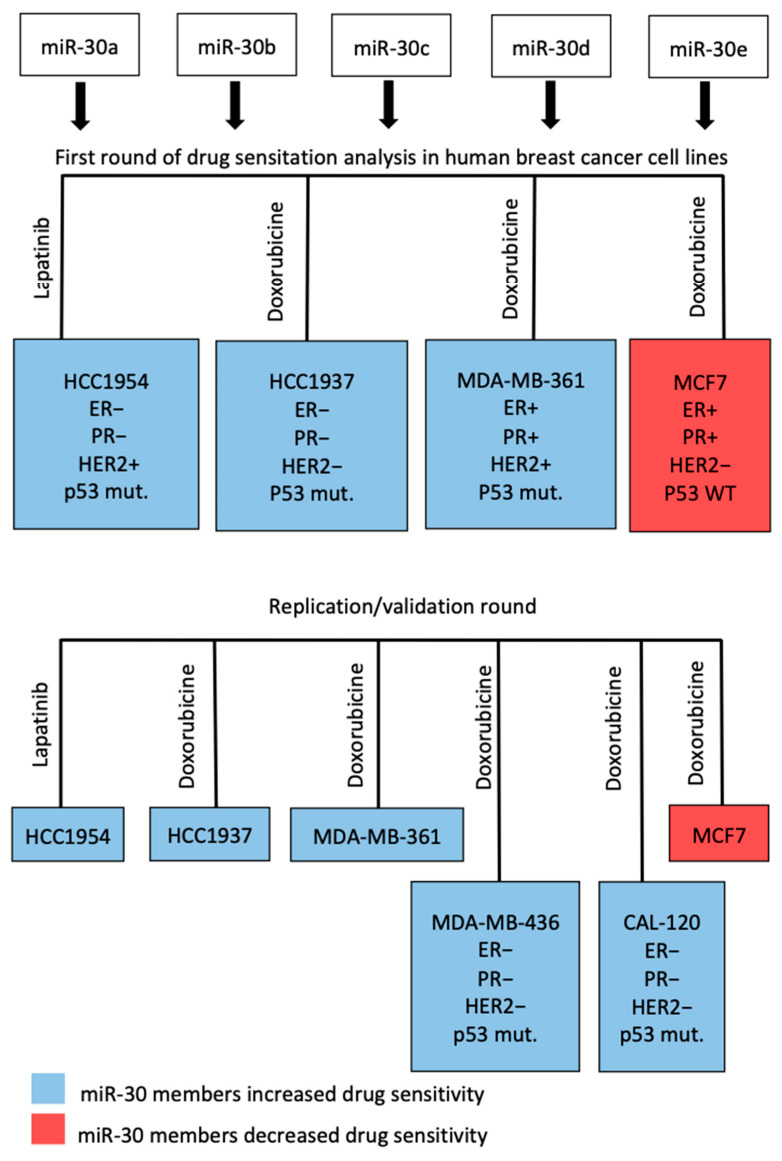
Diagram of the workflow of the drug-screening.

We investigated the effect of miR-30 family members on response to doxorubicin, an anthracycline-based chemotherapy, in multiple human breast cancer cell lines: HCC1937, MDA-MB-361, MCF7, and on response to lapatinib, a dual inhibitor of HER2, in HER2-positive HCC1954. Figure 3 illustrates the workflow of the drug sensitization in the primary screen (6 replicates for each miR-30 member) and replication round (11 replicates each), which included two additional cell lines, MDA-MB-436 and CAL-120, and all the above-mentioned cell lines. Transfection for drug sensitivity screening experiments with miR-30 family member mimics (i.e., miR-30a–e_mimics), and their specific inhibitors (i.e., miR-30a–e_anti) were carried out in all of the studied cell lines. To quantify the response to drugs, we calculated a drug sensitivity score (DSS) [27,28,29] based on miR-30-exposed cell viability at increasing drug concentrations.

**Figure 4:**
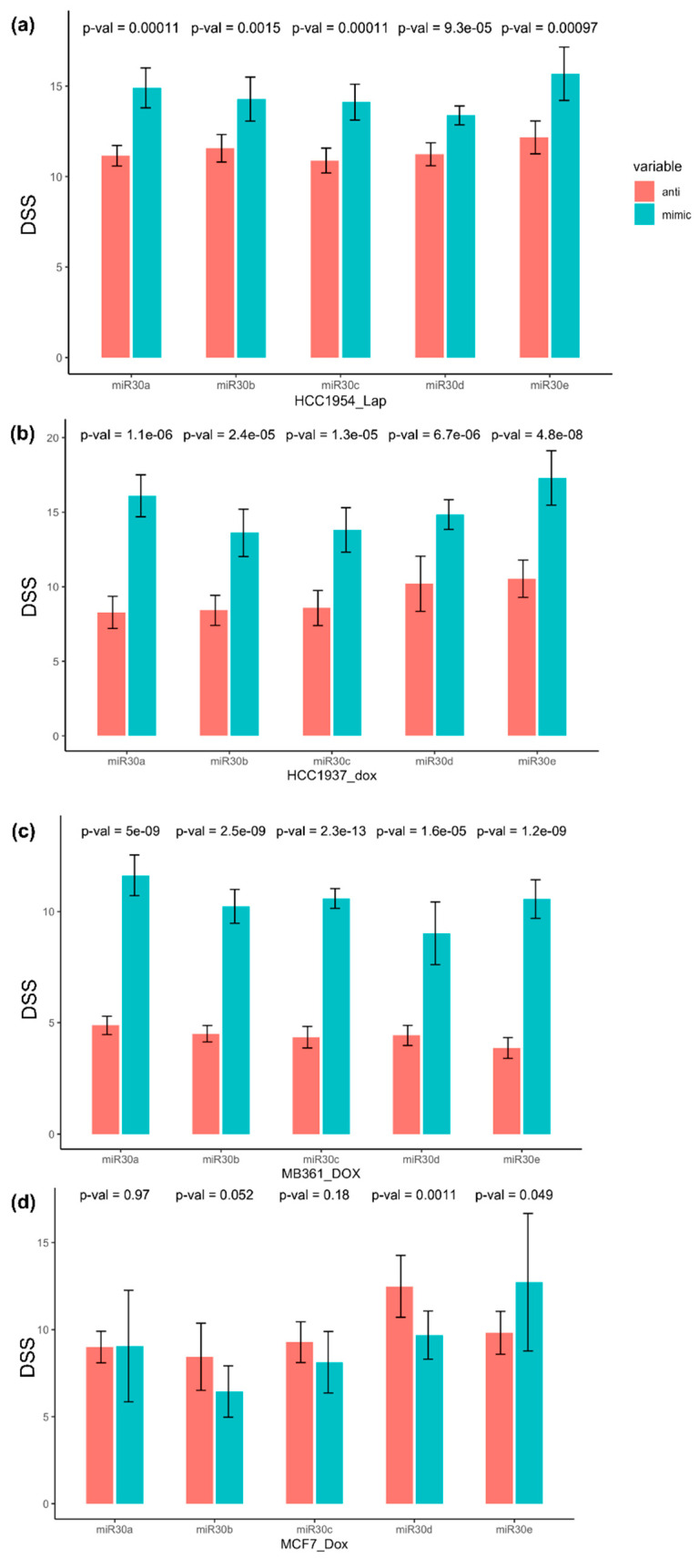
First round of drug screening test: compared to their inhibitors (red/left bars), miR-30 family member-specific mimics (blue/right bars) sensitized breast cancer cell line (**a**) HCC1954 to lapatinib, and (**b**) HCC1937, (**c**) MDA-MB-361, and (**d**) MCF7 to doxorubicin. Higher DSS (drug sensitivity score) indicates higher sensitivity to the drug. Lap: Lapatinib, Dox: Doxorubicin.

The HER2-positive HCC1954 cell line was strongly sensitized to HER2-targeting lapatinib when transfected with miR-30 family member mimics, compared to the inhibitors (largest *p* = 0.001) (Figure 4a, Table S4). The association was reproduced in the replication round with the largest *p* = 0.012. Table S4 and Figure S3A illustrate the comparison of drug sensitivity scores for cells transfected with miR-30 member mimics and their corresponding inhibitors in the replication round.

In the doxorubicin-response analysis, all miR-30 mimics strongly sensitized the cells to doxorubicin (higher DSS) in the triple-negative HCC1937, compared to miR-30 family member inhibitors, with the largest *p* < 10^−4^ (Figure 4b, Table S4). The effect was reproduced in the replication round (largest *p* < 10^−6^) (Table S4, Figure S3B). A similar effect was seen in the Luminal-like, HER2-positive MDA-MB-361 cells for all miR-30 members in the primary (largest *p* < 10^−4^) (Figure 4c, Table S4) and replication (largest *p* < 10^−6^) round (Table S4, Figure S3C).

In the primary screen of the Luminal-like, HER2-negative MCF7 cell line, we observed an opposite association between the miR-30 members and doxorubicin sensitivity. The miR-30-mimics appeared to increase the drug resistance (lower DSS), whereas the miR-30-anti showed elevated drug sensitivity with *p*-values varying between 0.971 and 0.001 (Figure 4d, Table S4). In the replication screen with a higher number of replicates, this trend was emphasized, with p-values varying between 0.11 and 10^−5^ for miR-30a, miR-30b, miR-30c, and miR-30d (Table S4, Figure S3D).

To validate our findings, we added the Basal-like MDA-MB-436 and CAL-120 cell lines to the replication round of drug sensitivity screening. All miR-30 member mimics increased doxorubicin sensitivity in MDA-MB-436 (largest *p* < 10^−14^) and CAL-120 (largest *p* < 10^−5^) cells, compared to the corresponding inhibitors (Table S4, Figure S3E,F, respectively).

### 3.4. The Putative Targets of the miR-30 Family Members in Breast Cancers Are Associated with Cellular Growth, Proliferation, and Motility

To identify the cellular functions and pathways which are affected by elevated mir-30 family expression in breast tumors, we used the METABRIC data for mRNA and miRNA expression in 1302 breast tumors [25] and IPA with two parallel approaches: the *microRNA Target Filter* analysis and the *Core* analysis. For the former, we included only the negatively correlated genes, since the miRNA function is assumed to be repressive of mRNA-level. In the *Core* analysis, we included all correlated genes, attempting to characterize which cellular pathways are active in tumors where the miR-30 family miRNAs have high expression. For this purpose, we used the Spearman correlation coefficients as proxies for log–fold change.

The top pathways, which emerged as significantly associated with all the miR-30 family members in both the *miRNA Target Filter* and the *Core* analyses, involved cell migration, motility, and cytoplasmic development (Table S5).

## 4. Discussion

We applied miRNA in situ hybridization to study the expression of miR-30d in human breast cancer tumors, and its association to tumor clinico-pathological characteristics and patient survival. We further explored the effects of miR-30 family members on drug response (doxorubicin and lapatinib) in vitro. We found that while high expression of miR-30d as such was a marker of aggressive tumor phenotype, i.e., higher grade, high proliferation rate, and positive nodal status, it was associated with improved patient survival. This favorable survival effect was enhanced in a multivariate survival analysis adjusted for the conventional prognostic factors, indicating that high expression of miR-30d is an independent predictor of better survival.

The miR-30d association with patient survival was especially pronounced in the subgroup of patients with HER2-positive tumors (Figure 2). Given that TOP2A amplification almost exclusively occurs in HER2-amplified breast cancers and that TOP2A is the primary target of doxorubicin [30,31], we speculate as to the existence of a miR-30/HER2/TOP2A connection, which may result in increased sensitivity to doxorubicin treatment and subsequently to better survival. Consistently, in the subgroup of patients who received chemotherapy, high miR-30d expression was associated with improved survival. The effect may, however, also be mediated by the high proliferation per se. The miR-30 family members also strongly sensitized the HER2-positive HCC1954 cell line to lapatinib, which targets HER2.

Virtually all miR-30 family member mimics sensitized the cells to doxorubicin when compared to their inhibitors. Earlier studies have demonstrated that miR-30 family members decrease chemo resistance in multiple cancers, including breast [32,33] and ovarian [34]. Here, the drug sensitization pattern was consistent in all our cell lines except for MCF7, in which miR-30 family mimics decreased doxorubicin sensitivity in comparison to their inhibitors. We postulate that the observed opposite effect in MCF7 might be associated with p53 status, as MCF7 was the only p53-proficient cell line in the panel. In the five other cell lines, which were p53-deficient, luminal and basal-like, high miR-30 sensitized the cells to doxorubicin. Consistently, in our survival analysis, higher miR-30d was associated with better survival among patients with p53-positive (mutated) tumors, and in chemotherapy-treated patients. Neither the *TP53* mRNA-expression, nor p53 IHC values were available in the METABRIC data to analyze any causal relationship between high miR-30 miRNA expression and p53 activity in these breast tumors. Previously, Guo et al. reported that forced expression of miR-30a repressed cancer cell motility and invasion in in vitro cell line constructs with *TP53* gain-of-function mutation R273H [35]. Furthermore, Di Gennaro et al. [36,37] showed that high endogenous miR-30a expression was associated with improved survival of triple-negative breast cancer patients, and validated miR-30a as a direct target of p53 transcriptional activity. They showed that p53 inhibits the expression of ZEB2, a transcription factor involved in EMT, through miR-30a to control tumor cell invasion and spreading. Our pathway enrichment analyses suggested an association for miR-30 family members and cell mobility, which may suggest a more general mechanism behind the observed effects. It can be postulated that the miR-30 family might function as an anti-metastatic factor in highly proliferating tumors: as long as the cells are proliferating, they remain immotile and do not invade. Other independent studies have also indicated the contribution of the miR-30 family to the regulation of cell proliferation, cell invasion, and EMT [38,39], particularly through their interaction with SNAI1/2 [40,41], metadherin (MTDH) [42], Slug, or TWIF1 [33,39]. The EMT process has been suggested to be associated with breast cancer chemo resistance [33,43,44]. However, the exact underlying mechanism remains to be further elucidated.

In contrary to the study by Li et al. [11], which suggested an inverse association between miR-30d expression and ovarian cancer patient survival, our clinical findings in breast cancer patients suggest that high miR-30d associates with improved survival. This is in line with reports of an independent survival study in the Genomic Data Common data portal miRNA-Seq dataset and The Cancer Genome Atlas (TCGA) with 1052 samples which reported that mir-30 family members, and particularly miR-30a, have significantly lower expression in breast cancers, and its high expression associates with better overall survival [45]. It seems that the direction in which miR-30 family members contribute to cancer aggressiveness, prognosis, and metastasis might be context-dependent and cancer-specific, as observed in other candidate miRNA studies [45,46]. For instance, while miR-30 family members appear to be anti-metastatic in breast cancer and non-small cell lung cancer [40], in melanoma cells and in hepatocellular carcinomas (HCC) the miR-30 family was pro-metastatic [47,48]. Similar to the tumor-suppressor miR-34a [46], the high expression of miR-30d was more often observed among the highly proliferating tumors. Whether miR-30d exerts its speculated anti-metastatic role particularly in proliferative tumors, and what the underlying mechanism is, warrant further investigation. In addition to their apparent cancer-specific roles, the different, and sometimes opposite, impacts of members of the miR-30 family on tumorigenesis and metastasis might be attributable to their diverse compensatory sequence, in spite of their sharing the same seed region.

To the best of our knowledge, this is the first extensive in vitro drug screening study for the miR-30 family in breast cancer. However, our study is limited by the magnitude of miRNAs’ potency in general, meaning that unlike in siRNA knockdown experiments, the miRNAs’ modest impact on target mRNA and protein level presents an experimental challenge when investigating their effects [49]. Nevertheless, the postulated connection between miR-30’s effects on drug sensitivity through HER2/TOP2A axis or p53 and cell migration connections can be viewed as hypothesis generating.

## 5. Conclusions

Taken together, our results suggest that miR-30d expression may provide a prognostic and predictive factor for breast cancer patients. Whether the observed association of high miR-30d expression and better clinical outcomes is due to its plausible connection with the doxorubicin, TOP2A, and p53 signaling pathways, or to its postulated anti-metastatic function, remains to be further studied.

## Figures and Tables

**Table 1:** Association of miR-30d expression with the clinical and pathological features of the tumors.

Category	miR-30d n (%)			*p*
	Total	Low ^1^	High ^1^	
Tumor histology				
Ductal	838 (70.2%)	233 (64.5%)	605 (72.7%)	0.003 ^2^
Lobular	222 (18.6%)	90 (24.9%)	132 (15.9%)	
Medullary	15 (1.3%)	3 (0.8%)	12 (1.4%)	
Other	118 (9.9%)	35 (9.7%)	83 (10.0%)	
Grade				
1	281 (23.9%)	96 (27.0%)	185 (22.5%)	0.0002
2	547 (46.5%)	183 (51.5%)	364 (44.3%)	
3	349 (29.7%)	76 (21.4%)	273 (33.2%)	
Tumor size				
1	694 (58.9%)	213 (60.2%)	481 (58.3%)	0.417
2	410 (34.8%)	115 (32.5%)	295 (35.8%)	
3	40 (3.4%)	16 (4.5%)	24 (2.9%)	
4	35 (3.0%)	10 (2.8%)	25 (3.0%)	
Nodal status ^3^				
Negative	645 (54.9%)	214 (61.0%)	431 (52.4%)	0.007
positive	529 (45.1%)	137 (39.0%)	392 (47.6%)	
M ^4^				
Negative	1147 (97.0%)	347 (96.9%)	800 (97.0%)	1.000
Positive	36 (3.0%)	11 (3.1%)	25 (3.0%)	
ER status				
Negative	234 (20.6%)	70 (21.0%)	164 (20.5%)	0.872
Positive	900 (79.4%)	263 (79.0%)	637 (79.5%)	
PR status				
Negative	379 (33.5%)	124 (37.0%)	255 (32.0%)	0.113
Positive	753 (66.5%)	211 (63.0%)	542 (68.0%)	
p53 tumor status				
Negative	899 (80.1%)	264 (82.8%)	635 (79.0%)	0.160
Positive	224 (19.9%)	55 (17.2%)	169 (21.0%)	
HER2 status				
Negative	984 (87.1%)	298 (90.0%)	686 (85.9%)	0.064
Positive	146 (12.9%)	33 (10.0%)	113 (14.1%)	
Ki-67 (proliferation marker)				
Negative	786 (67.2%)	250 (72.5%)	536 (65.0%)	0.014
Positive	383 (32.8%)	95 (27.5%)	288 (35.0%)	
ER/PR/HER2 status ^5^				
ER/PR+ and HER2−	778 (72.6%)	232 (76.1%)	546 (71.2%)	0.032
ER/PR+ and HER2+	91 (8.5%)	14 (4.6%)	77 (10.0%)	
ER/PR− and HER2+	52 (4.9%)	17 (5.6%)	35 (4.6%)	
ER− and PR− and HER2−	151 (14.1%)	42 (13.8%)	109 (14.2%)	

^1.^ Low: None or weak cytoplasmic staining, High: moderate or high cytoplasmic staining. ^2.^ Fisher’s exact test. ^3.^ Nodal status: development of nodal metastasis at diagnosis. ^4.^ Metastasis at diagnosis. ^5.^ The + denotes positive status and the − denotes negative status.

**Table 2:** High expression of miR-30d emerged as independent prognostic factor in the multivariate Cox regression model for 5-year BDDM, and 10-year BCS analyses.

Covariate	5-Year BDDM			10-Year BCS		
	*p*	HR	95%CI	*p*	HR	95%CI
Grade	0.0001	2.28	1.45–3.49	0.0004	2.06	1.37–3.09
T	1.7 × 10^−6^	1.58	1.24–2.01	0.001	1.50	1.17–1.93
N	1.1 × 10^−6^	2.95	1.77–4.91	1.9 × 10^−5^	3.11	1.84–5.23
M	-	-	-	5.8 × 10^−8^	6.31	3.24–12.2
ER	0.60	1.16	0.66–2.045	0.50	0.82	0.46–1.45
Ki67	0.97	0.97	0.57–1.71	0.70	0.90	0.55–1.48
HER2	0.38	1.62	0.95–2.78	0.17	1.48	0.85–2.57
p53	0.44	1.32	0.77–2.25	0.23	1.37	0.81–2.32
miR-30d	0.0004	0.44	0.28–0.69	0.006	0.54	0.35–0.84

## Data Availability

The drug sensitivity score and relevant statistical data required for study replication of the drug-sensitivity analyses are available in Table S4. The samples’ survival datasets used and/or analyzed during the current study are available from the corresponding author on reasonable request. Code availability: The analyses were conducted in R, thus, all of the statistical packages used here are publicly available. The codes are available from the corresponding author upon request.

## Supplementary Materials

The following are available online at https://www.mdpi.com/article/10.3390/cancers13122907/s1, Figure S1: Samples of tumor microarray slides, Figure S2: Flow chart of the sample inclusion criteria in various statistical analyses, Figure S3: Replication round of drug screening test, Table S1: List of the specific miRNA-mimics and miRNA-inhibitors for each miR-30 family member, Table S2: Association of miR-30d expression with the clinical and pathological features of tumors of incident cases, Table S3: Univariate Cox’s regression analysis by miR-30d, Table S4: Applying drug sensitivity score (DSS) to compare miR-30 family member mimics with their inhibitors using Student’s *t*-test, Table S5: Results of the Ingenuity Pathway Analysis of genes correlated with the expression of the miR-30 family miRNAs.
