# Supplementary material for: High miR-30 Expression Associates with Improved Breast Cancer Patient Survival and Treatment Outcome
Source: Cancers (Basel). 2021 Jun 10;13(12):2907. doi: 10.3390/cancers13122907 (PMC8230388; doi:10.3390/cancers13122907)
Supplement: Supplementary file 1 [file cancers-13-02907-s001.zip › cancers-1256527-supplementary.pdf]

cancers

MDPI

High miR-30 Expression Associates with Improved Breast Cancer Patient Survival and Treatment Outcome

Maral Jamshidi, Rainer Fagerholm, Taru A. Muranen, Sippy Kaur, Swapnil Potdar, Sofia Khan, Eliisa Netti, John-Patrick Mpindi, Bhagwan Yadav, Johanna I. Kiiski, Kristiina Aittomäki, Päivi Heikkilä, Jani Saarela, Ralf Bützow, Carl Blomqvist and Heli Nevanlinna

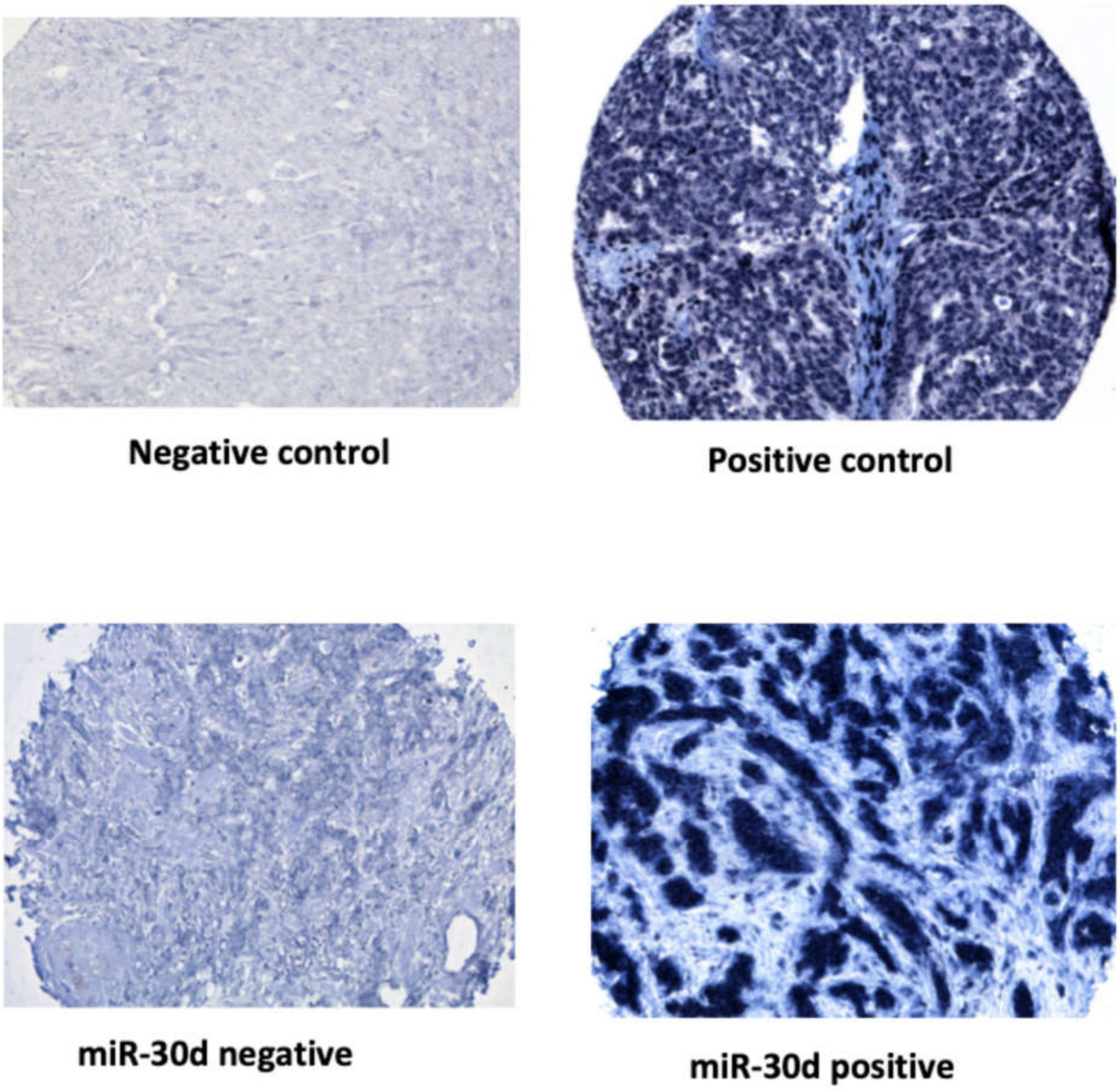

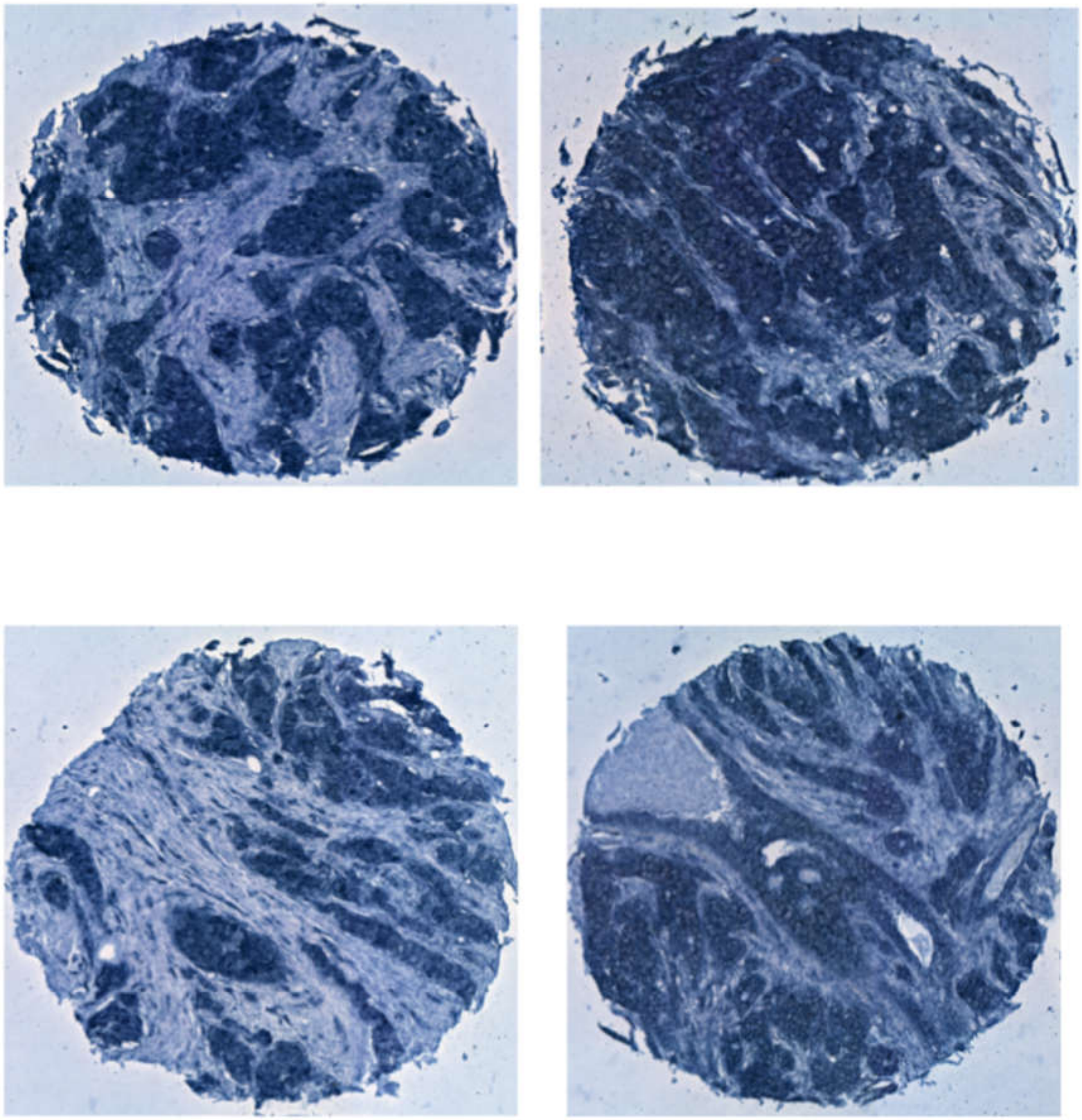

miR-30d positive (high expression)

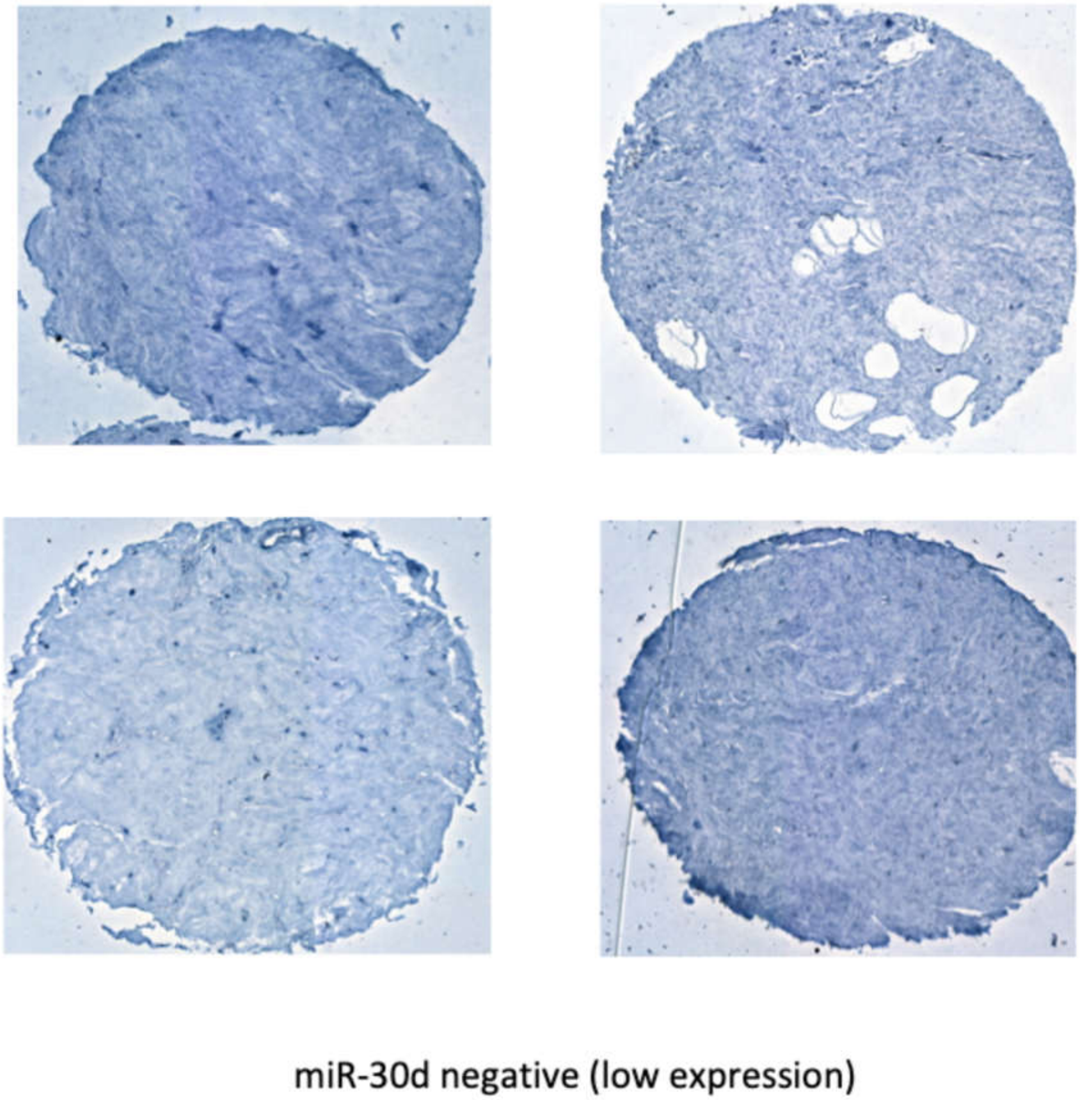

Figure S1 Samples of tumor microarray slides.

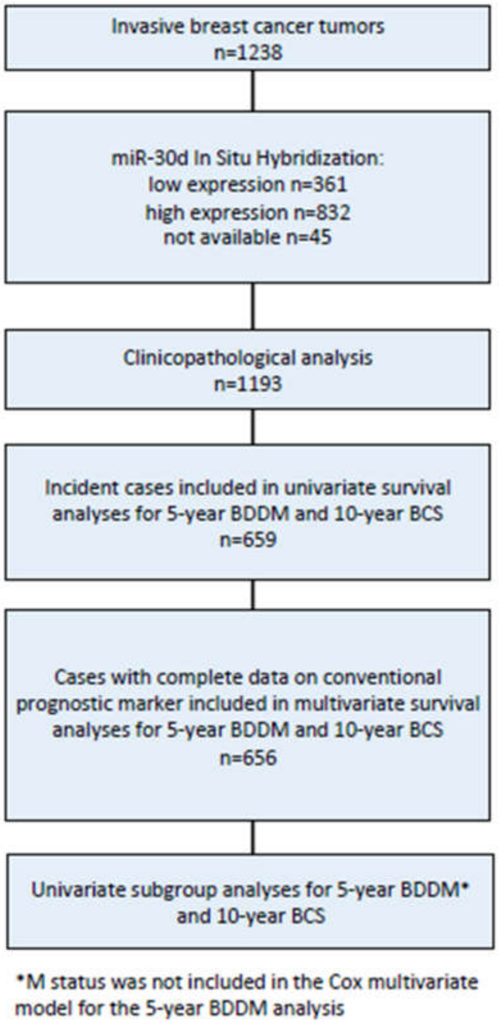

Figure S2: Flow chart of the samples' inclusion criteria in various statistical analyses.

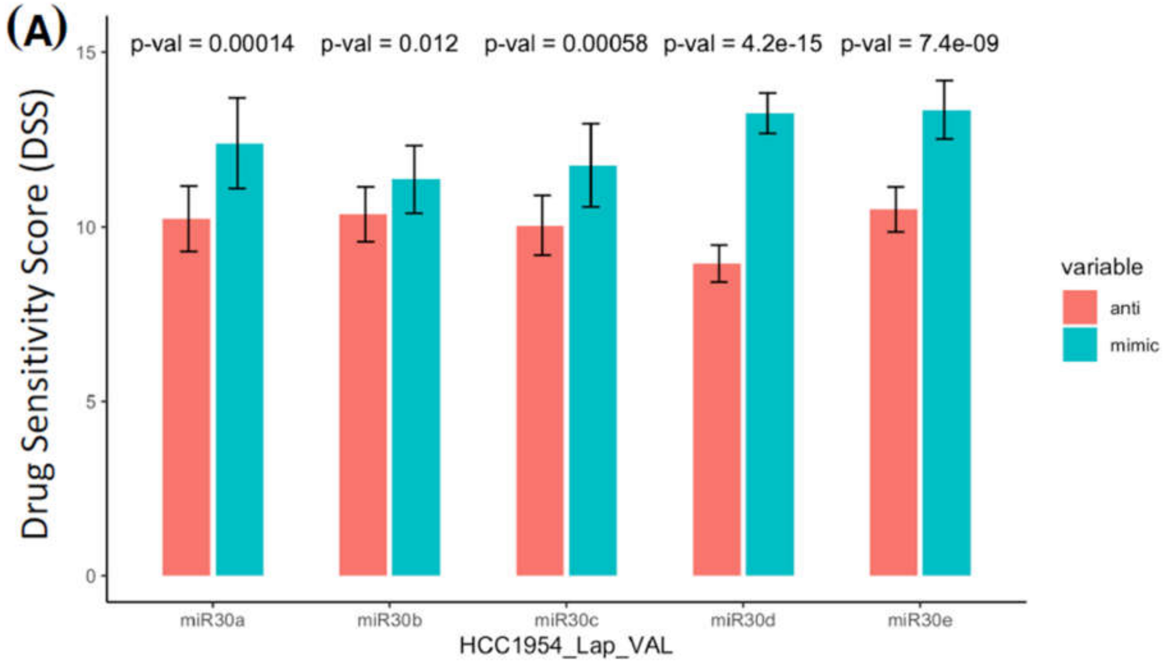

HCC1954 cell lines treated with lapatinib

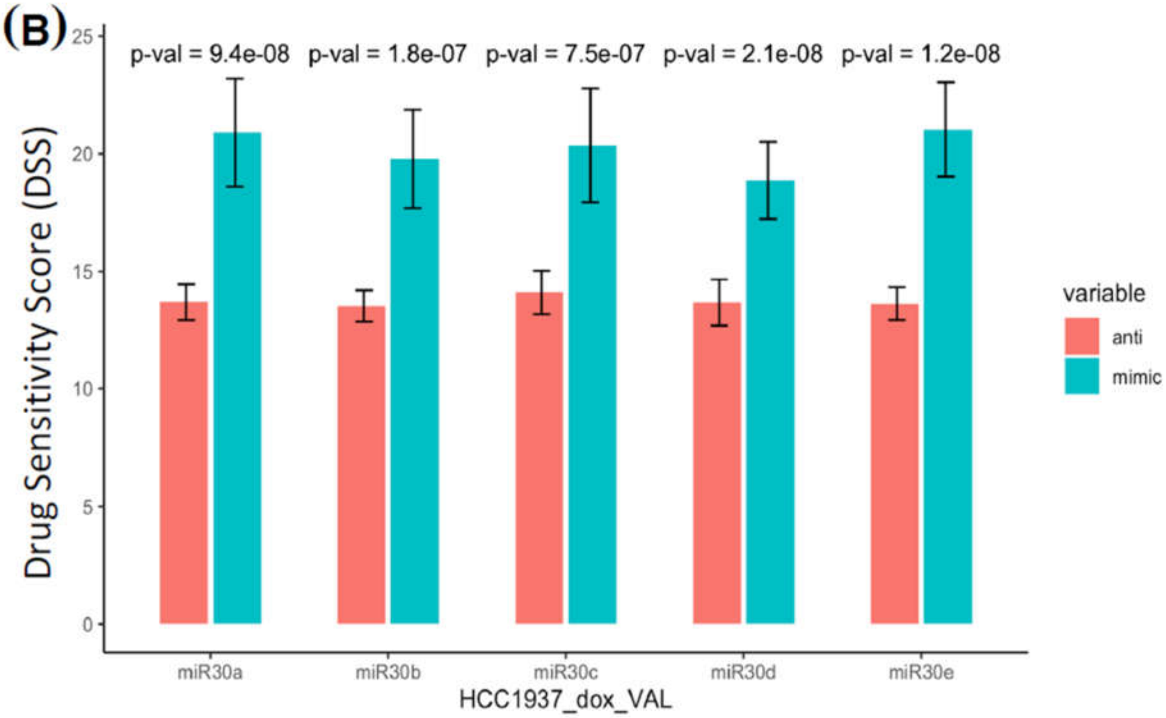

HCC1937 cell lines treated with doxorubicin

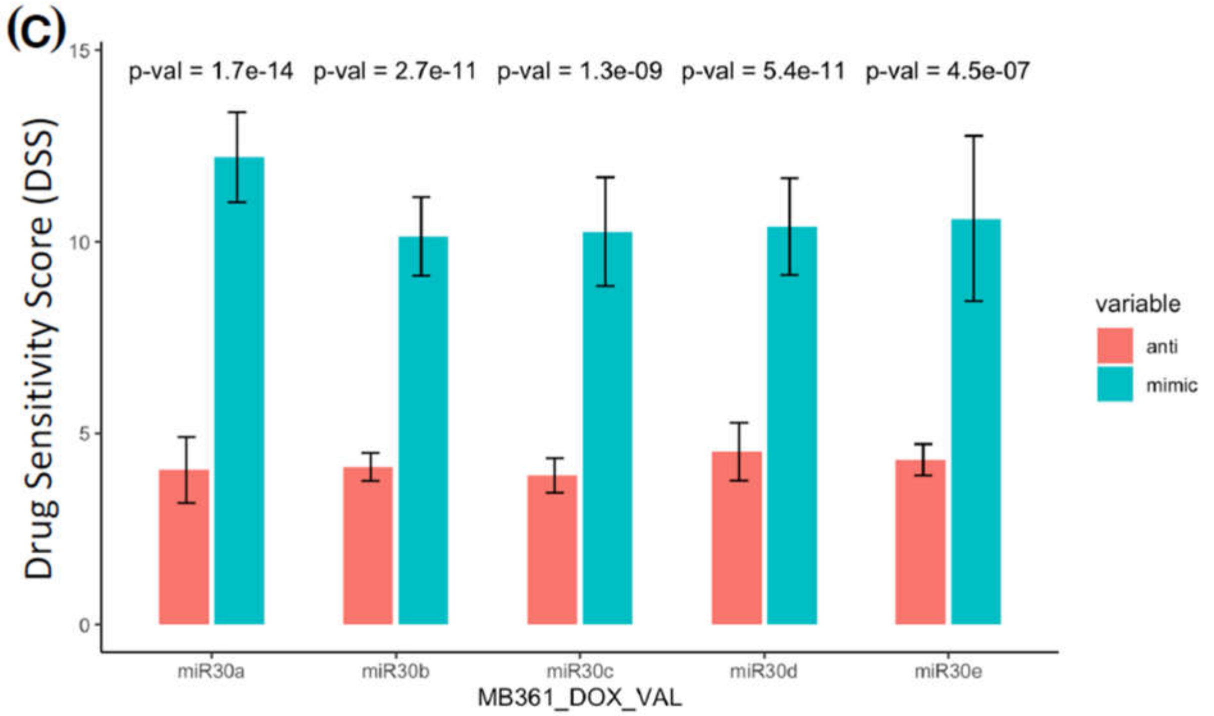

MDA-MB-361 cell lines treated with doxorubicin

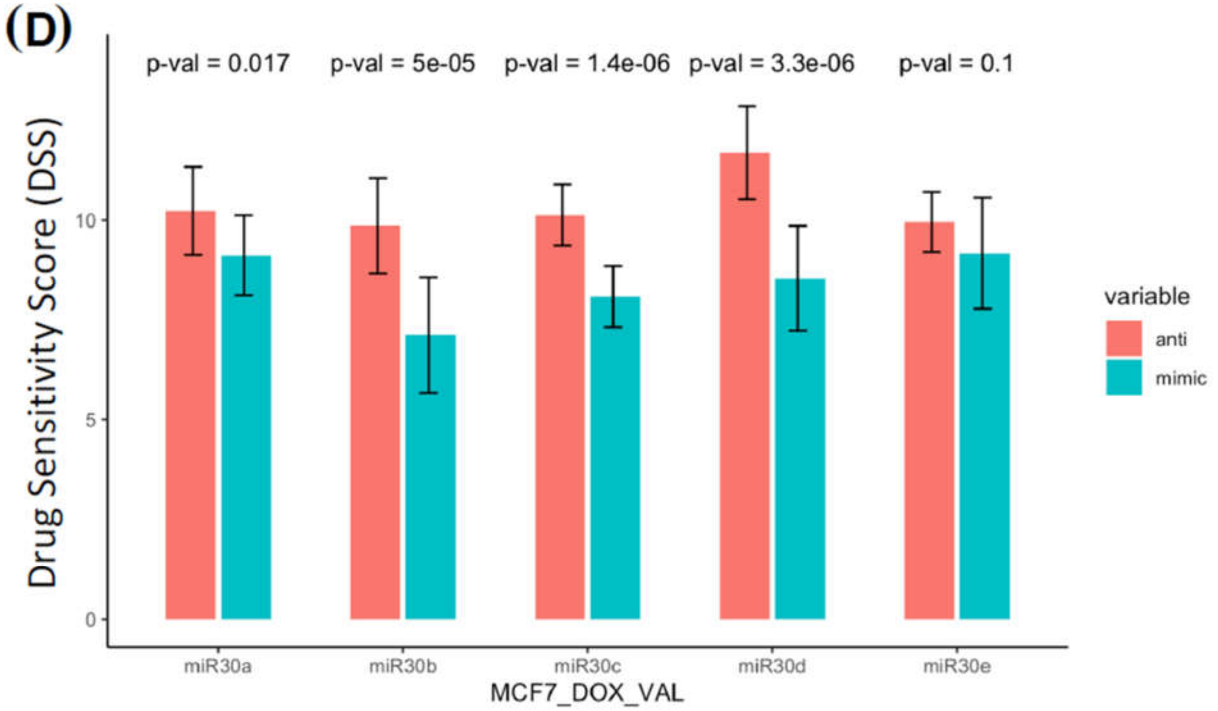

MCF7 cell lines treated with doxorubicin

(E)

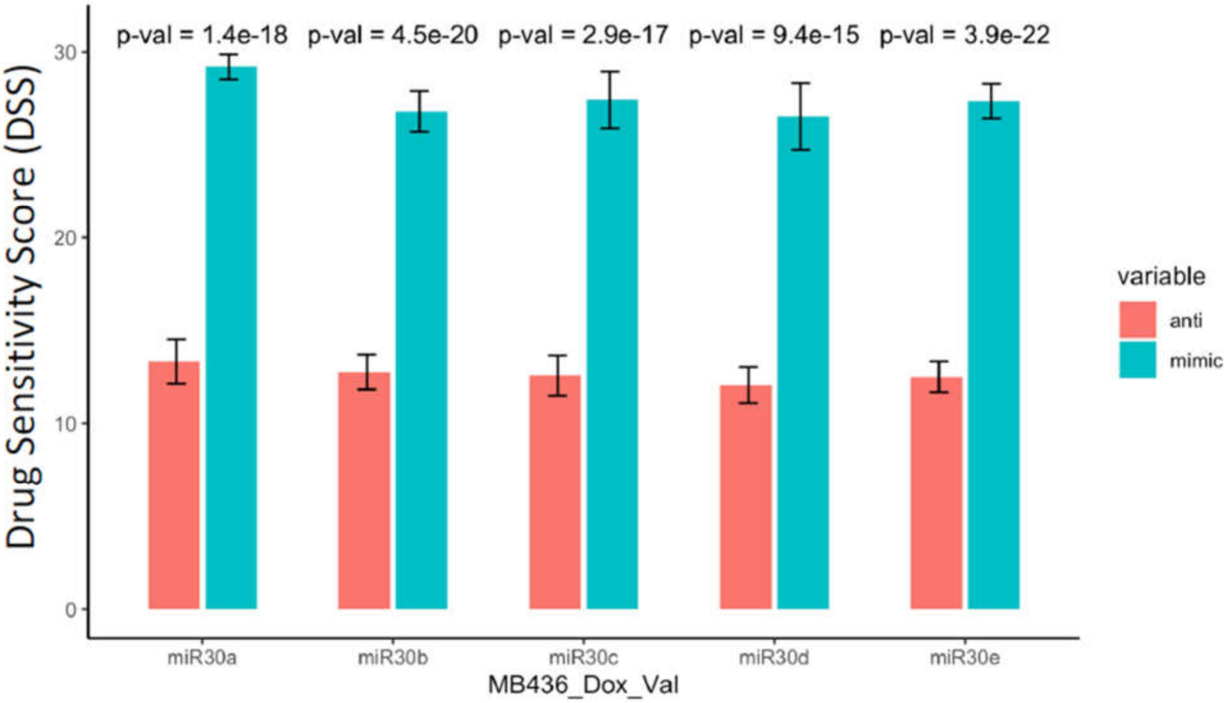

MDA-MB-436 cell lines treated with doxorubicin

(F)

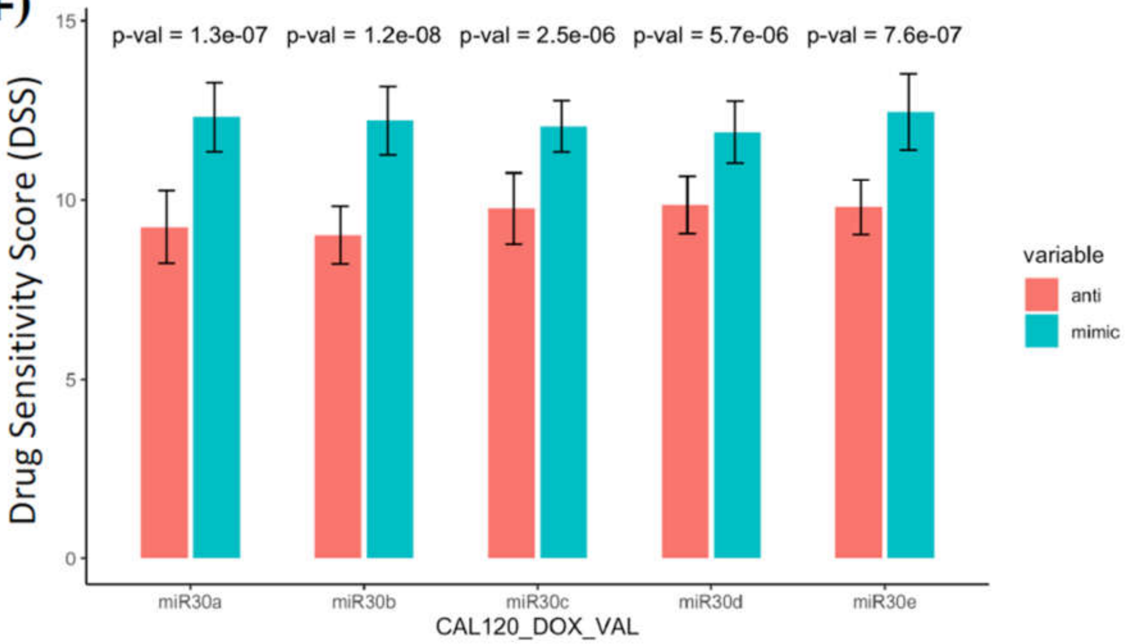

CAL120 cell lines treated with doxorubicin

Figure S3. Replication round of drug screening test: DSS for cells transfected with miR-30 family member-specific mimics and inhibitors. Compared to their inhibitors, the miR-30 mimics sensitized the breast cancer cell lines to doxorubicin and lapatinib. Higher DSS score indicates increased drug sensitivity. Dox: doxorubicin, Lap: Lapatinib, VAL: validation, MB361: MDA-MB-361, MB436: MDA-MB-436 cell line.

Table S1. List of the specific miRNA-mimics and miRNA-inhibitors for each miR-30 family member.

miRNA	Product type	Ambion ID	Mature miRNA Sequence
hsa-miR-30a-5p	mirVana miRNA mimic	MC11062	UGUAAACAUCCUCGACUGGAAG
hsa-miR-30a-5p	mirVana miRNA inhibitor	MH11062	UGUAAACAUCCUCGACUGGAAG
hsa-miR-30b-5p	mirVana miRNA mimic	MC10986	UGUAAACAUCCUACACUCAGCU
hsa-miR-30b-5p	mirVana miRNA inhibitor	MH10986	UGUAAACAUCCUACACUCAGCU
hsa-miR-30c-5p	mirVana miRNA mimic	MC11060	UGUAAACAUCCUACACUCUCAGC
hsa-miR-30c-5p	mirVana miRNA inhibitor	MH11060	UGUAAACAUCCUACACUCUCAGC
hsa-miR-30d-5p	mirVana miRNA mimic	MC10756	UGUAAACAUCCCCGACUGGAAG
hsa-miR-30d-5p	mirVana miRNA inhibitor	MH10756	UGUAAACAUCCCCGACUGGAAG
hsa-miR-30e-5p	mirVana miRNA mimic	MC10037	UGUAAACAUCCUUGACUGGAAG
hsa-miR-30e-5p	mirVana miRNA inhibitor	MH10037	UGUAAACAUCCUUGACUGGAAG

Table S2. Association of miR-30d expression with the clinical and pathological features of tumors of incident cases. (n, %)

	Total	**miR-30d** ***Low***	**miR-30d** ***High***	***p***
Tumour histology				
Ductal carcinomas	452	125	327	0.008
	68.6%	61.0%	72.0%	
Lobular carcinomas	126	54	72	
	19.1%	26.3%	15.9%	
Medullary carcinomas	7	1	6	
	1.1%	.5%	1.3%	
Other	74	25	49	
	11.2%	12.2%	10.8%	
Grade				
1	154	57	97	0.008
	23.5%	28.2%	21.5%	
2	295	98	197	
	45.1%	48.5%	43.6%	
3	205	47	158	
	31.3%	23.3%	35.0%	
Tumor size				
1	370	119	251	0.264
	56.4%	58.3%	55.5%	
2	243	68	175	
	37.0%	33.3%	38.7%	
3	21	10	11	
	3.2%	4.9%	2.4%	
4	22	7	15	
	3.4%	3.4%	3.3%	
Nodal status (development of nodal metastasis at diagnosis)				
Negative	335	115	220	0.063
	51.2%	56.7%	48.8%	
positive	319	88	231	
	48.8%	43.3%	51.2%	
Metastasis at diagnosis (development of distant metastasis at diagnosis)				
Negative	633	196	437	0.660
	96.2%	95.6%	96.5%	
Positive	25	9	16	
	3.8%	4.4%	3.5%	
ER status				
Negative	127	36	91	0.522

	19.6%	18.0%	20.3%	
Positive	521	164	357	
	80.4%	82.0%	79.7%	
PR status				
Negative	220	73	147	0.419
	33.9%	36.3%	32.8%	
Positive	429	128	301	
	66.1%	63.7%	67.2%	
p53 tumor status				
Negative	497	155	342	0.080
	79.6%	84.2%	77.7%	
Positive	127	29	98	
	20.4%	15.8%	22.3%	
HER2 status				
Negative	541	170	371	0.093
	86.8%	90.4%	85.3%	
Positive	82	18	64	
	13.2%	9.6%	14.7%	
Ki67 tumor status (proliferation marker)				
Negative	398	135	263	0.023
	61.1%	67.8%	58.2%	
Positive	253	64	189	
	38.9%	32.2%	41.8%	
SubTypes				
ER/PR pos and HER2 neg	453	147	306	0.054
	73.9%	79.9%	71.3%	
ER/PR pos and HER2 pos	47	7	40	
	7.7%	3.8%	9.3%	
ER/PR neg and HER2 pos	35	11	24	
	5.7%	6.0%	5.6%	
ER neg and PR neg and HER2 neg	78	19	59	
	12.7%	10.3%	13.8%	

Low=None or weak cytoplasmic staining, High=moderate or high cytoplasmic staining.

Table S3. Univariate Cox's regression analysis by miR-30d in all patients, and in patients subgrouped by ER, HER2, Ki67, p53, and chemotherapy status for (**A**) 5-years BDDM (breast cancer death or distant metastasis), and (**B**) 10-year BCS (breast cancer survival) among incident cases.

**Category**	**5-year BDDM**				
	**miR-30d n(event)***		**p value**	**HR**	**95% CI**
	**Low**	**High**			
**A)**					
**All patients**	205 (45)	451 (66)	0.019	0.63	0.44-0.93
**ER-negative**	36 (16)	89 (18)	0.006	0.38	0.19-0.76
**ER-positive**	164 (29)	356 (48)	0.208	0.74	0.46-1.18
**HER2-negative**	170 (32)	371 (50)	0.107	0.68	0.44-1.07
**HER2-positive**	18 (12)	61 (15)	0.001	0.27	0.12-0.59
**Ki67-negative**	135 (19)	261 (32)	0.598	0.85	0.48-1.51
**Ki67-positive**	64 (26)	188 (34)	0.0002	0.38	0.23-0.64
**p53-negative**	155 (29)	340 (44)	0.106	0.67	0.42-1.07
**p53-positive**	29 (13)	97 (21)	0.011	0.41	0.20-0.81

**No chemotherapy**	131 (22)	254 (32)	0.285	0.74	0.43-1.28
**Chemotherapy**	74 (23)	196 (34)	0.011	0.49	0.29-0.84
**No-Anthracycline**	32 (8)	102 (17)	0.246	0.61	0.26-1.41
**Anthracycline**	42 (15)	94 (17)	0.023	0.44	0.22-0.89
**B)**			**10-year BCS**		
**All patients**	205 (41)	451 (58)	0.018	0.61	0.41-0.92
**ER-negative**	36 (14)	89 (17)	0.015	0.41	0.20-0.84
**ER-positive**	164 (27)	356 (41)	0.126	0.68	0.46-1.11
**HER2-negative**	170 (29)	371 (45)	0.197	0.68	0.42-1.08
**HER2-positive**	18 (11)	61 (12)	0.0006	0.24	0.10-0.54
**Ki67-negative**	135 (20)	261 (30)	0.291	0.73	0.41-1.29
**Ki67-positive**	64 (21)	188 (28)	0.002	0.41	0.23-0.73
**p53-negative**	155 (26)	340 (37)	0.075	0.62	0.37-1.03
**p53-positive**	29 (11)	97 (20)	0.043	0.46	0.22-0.97
**No chemotherapy**	131 (22)	254 (27)	0.112	0.63	0.36-1.11
**Chemotherapy**	74 (19)	196 (31)	0.045	0.55	0.31-0.98
**No-Anthracycline**	32 (8)	102 (17)	0.255	0.61	0.26-1.42
**Anthracycline**	42 (11)	94 (14)	0.131	0.54	0.24-1.21

Table S4. Applying Drug Sensitivity Score (DSS) to compare miR-30 family member mimics with their inhibitors using student *t*-test.

Screening round	cell line	Type	Treatment	miR30 member	p_value	stderr	lower 95%CI	upper 95%CI
First	HCC1954	HER2pos	Lapatinib	miR30a	1.08E-04	0.51	2.57	4.93
First	HCC1954	HER2pos	Lapatinib	miR30b	1.08E-04	0.51	2.57	4.93
First	HCC1954	HER2pos	Lapatinib	miR30c	1.10E-04	0.49	2.12	4.35
First	HCC1954	HER2pos	Lapatinib	miR30d	9.34E-05	0.34	1.4	2.9
First	HCC1954	HER2pos	Lapatinib	miR30e	9.68E-04	0.71	1.9	5.14
Second	HCC1954	HER2pos	Lapatinib	miR30a	1.38E-04	0.46	1.2	3.13
Second	HCC1954	HER2pos	Lapatinib	miR30b	1.16E-02	0.36	0.25	1.75
Second	HCC1954	HER2pos	Lapatinib	miR30c	5.76E-04	0.42	0.85	2.62
Second	HCC1954	HER2pos	Lapatinib	miR30d	4.20E-15	0.23	3.84	4.78
Second	HCC1954	HER2pos	Lapatinib	miR30e	7.44E-09	0.3	2.21	3.48
First	HCC1937	Triple Neg	Doxorubicin	miR30a	1.07E-06	0.7	6.23	9.4
First	HCC1937	Triple Neg	Doxorubicin	miR30b	2.40E-05	0.71	3.61	6.79
First	HCC1937	Triple Neg	Doxorubicin	miR30c	1.26E-05	0.72	3.67	6.81
First	HCC1937	Triple Neg	Doxorubicin	miR30d	6.67E-06	0.66	3.22	6.06
First	HCC1937	Triple Neg	Doxorubicin	miR30e	4.82E-08	0.7	5.27	8.25
Second	HCC1937	Triple Neg	Doxorubicin	miR30a	9.36E-08	0.7	5.72	8.73
Second	HCC1937	Triple Neg	Doxorubicin	miR30b	1.78E-07	0.63	4.89	7.62
Second	HCC1937	Triple Neg	Doxorubicin	miR30c	7.47E-07	0.75	4.65	7.85
Second	HCC1937	Triple Neg	Doxorubicin	miR30d	2.15E-08	0.55	4.04	6.36
Second	HCC1937	Triple Neg	Doxorubicin	miR30e	1.15E-08	0.61	6.1	8.73
First	MB361	ER+PR+HER2+	Doxorubicin	miR30a	5.04E-09	0.36	5.97	7.56
First	MB361	ER+PR+HER2+	Doxorubicin	miR30b	2.50E-09	0.3	5.07	6.4
First	MB361	ER+PR+HER2+	Doxorubicin	miR30c	2.26E-13	0.23	5.74	6.74
First	MB361	ER+PR+HER2+	Doxorubicin	miR30d	1.63E-05	0.52	3.4	5.8
First	MB361	ER+PR+HER2+	Doxorubicin	miR30e	1.18E-09	0.35	5.93	7.47

Second	MB361	ER+PR+HER2+	Doxorubicin	miR30a	1.72E-14	0.42	7.29	9.05
Second	MB361	ER+PR+HER2+	Doxorubicin	miR30b	2.72E-11	0.32	5.35	6.7
Second	MB361	ER+PR+HER2+	Doxorubicin	miR30c	1.29E-09	0.43	5.44	7.29
Second	MB361	ER+PR+HER2+	Doxorubicin	miR30d	5.41E-11	0.43	4.99	6.78
Second	MB361	ER+PR+HER2+	Doxorubicin	miR30e	4.46E-07	0.63	4.92	7.68
First	MCF7	ER+PR+HER2-P53-wild	Doxorubicin	miR30a	9.65E-01	1.26	-2.93	3.04
First	MCF7	ER+PR+HER2-P5t-wild	Doxorubicin	miR30b	5.16E-02	0.92	-4.02	0.02
First	MCF7	ER+PR+HER2-P5t-wild	Doxorubicin	miR30c	1.77E-01	0.8	-2.93	0.62
First	MCF7	ER+PR+HER2-P5t-wild	Doxorubicin	miR30d	1.07E-03	0.71	-4.3	-1.3
First	MCF7	ER+PR+HER2-P5t-wild	Doxorubicin	miR30e	4.88E-02	1.31	0.02	5.8
Second	MCF7	ER+PR+HER2-P5t-wild	Doxorubicin	miR30a	1.68E-02	0.43	-2.01	-0.22
Second	MCF7	ER+PR+HER2-P5t-wild	Doxorubicin	miR30b	4.99E-05	0.54	-3.87	-1.62
Second	MCF7	ER+PR+HER2-P5t-wild	Doxorubicin	miR30c	1.42E-06	0.31	-2.7	-1.4
Second	MCF7	ER+PR+HER2-P5t-wild	Doxorubicin	miR30d	3.25E-06	0.51	-4.2	-2.1
Second	MCF7	ER+PR+HER2-P5t-wild	Doxorubicin	miR30e	1.02E-01	0.46	-1.76	0.18
Second	CAL 120	Triple Neg	Doxorubicin	miR30a	1.28E-07	0.4	2.24	3.91
Second	CAL 120	Triple Neg	Doxorubicin	miR30b	1.17E-08	0.36	2.45	3.95
Second	CAL 120	Triple Neg	Doxorubicin	miR30c	2.51E-06	0.36	1.57	3.05
Second	CAL 120	Triple Neg	Doxorubicin	miR30d	5.72E-06	0.34	1.32	2.74
Second	CAL 120	Triple Neg	Doxorubicin	miR30e	7.62E-07	0.38	1.88	3.46
Second	MB436	Triple Neg	Doxorubicin	miR30a	1.45E-18	0.4	15.02	16.7
Second	MB436	Triple Neg	Doxorubicin	miR30b	4.50E-20	0.42	13.17	14.9
Second	MB436	Triple Neg	Doxorubicin	miR30c	2.91E-17	0.54	13.71	15.97
Second	MB436	Triple Neg	Doxorubicin	miR30d	9.36E-15	0.59	13.23	15.7
Second	MB436	Triple Neg	Doxorubicin	miR30e	3.88E-22	0.36	14.08	15.57

Table S5. Results of the Ingenuity Pathway Analysis of genes correlated with the expression of the miR-30 family miRNAs.

	**miRNA target filter analysis**			**Core analysis**		**Notes on the METABRIC-IPA analysis**
	**n filtered target genes**	**Top disease**	**Top function**	**n correlated genes**	**Top function**	
**hsa-miR-30a-5p**	23	Carcinoma<br>23/23 target genes<br>p-value of overlap<br>3.2E-7	Migration of cells<br>9/23 target genes<br>p-value of overlap<br>2.2E-3	479<br>(313 positively<br>166 negatively)	Cell movement of breast cancer cell lines<br>27 genes<br>activation z-score -1.6<br>p-value of overlap 4.0E-6	
**hsa-miR-30a-3p**	31	Breast or gyneco-logical cancer<br>20/31 target genes<br>p-value of overlap<br>5.4E-7	Cell movement<br>16/31 target genes<br>p-value of overlap<br>1.3E-6	1975<br>(995 positively<br>980 negatively)	Cell movement - predicted de-creased<br>471 genes<br>activation z-score -5.0<br>p-value of overlap 2.0E-20	
**hsa-miR-30b-5p**	27	Abdominal carci-noma<br>26/27 target genes<br>p-value of overlap<br>2.6E-8	Cell movement<br>13/27 target genes<br>p-value of overlap<br>5.6E-5	568<br>(288 positively<br>280 negatively)	Cell movement - predicted de-creased<br>165 genes<br>activation z-score -4.8<br>p-value of overlap 9.1E-13	
**hsa-miR-30b-3p**	15	Epithelial neo-plasm<br>14/15 target genes<br>p-value of overlap<br>7.5E-4	Development of cytoplasm<br>4/15 target genes<br>p-value of overlap<br>6.1E-4	212<br>(114 positively<br>98 negatively)	Development of cytoplasm<br>16 genes<br>activation z-score -1.7<br>p-value of overlap 1.6E-3	A_25_P00013382 probe for hsa-miR-30b-3p has very low signal in the METABRIC, suggesting that this is the inactive strand with short half-life.
**hsa-miR-30c-5p**	67	Carcinoma<br>62/67 target genes<br>p-value of overlap<br>3.2E-13	Migration of cells<br>25/67 target genes<br>p-value of overlap<br>4.2E-7	1868<br>(867 positively<br>1001 negatively)	Migration of cells - predicted de-creased<br>409 genes<br>activation z-score -5.0<br>p-value of overlap 6.7E-20	
**hsa-miR-30c1-3p**	0			1<br>(1 positively<br>0 negatively)		A_25_P00013489 probe for hsa-miR-30c-1-3p has very low signal in the METABRIC, suggesting that this is the inactive strand with short half-life.

**hsa-miR-30c2-3p**	61	Carcinoma<br>57/61 target genes<br>p-value of overlap<br>9.4E-12	Cell movement<br>29/61 target genes<br>p-value of overlap<br>8.7E-10	1817<br>(916 positively<br>901 negatively)	Cell movement - predicted decreased<br>437 genes<br>activation z-score -5.0<br>p-value of overlap 1.0E-19	
**hsa-miR-30d-5p**	0			83<br>(83 positively<br>0 negatively)		A_25_P00010683 probe for hsa-miR-30d-5p has a good expression signal in the METABRIC. However, the probe sequence is a perfect match to a lncRNA transcript AC004263.1-201/ENST00000617183.1 and lncRNA transcript AL022318.1-201/ENST00000450216.1, which if expressed in the METABRIC tumors, may be an error source.
**hsa-miR-30d-3p**	0			121<br>(92 positively<br>29 negatively)		A_25_P00013291 probe for hsa-miR-30d-3p has very low signal in the METABRIC, suggesting that this is the inactive strand with short half-life.
**hsa-miR-30e-5p**	0			1<br>(1 positively<br>0 negatively)		A_25_P00012300 probe for hsa-miR-30e-5p and A_25_P00014611 probe for hsa-miR-30e-3p have both good expression signal in the METABRIC. The correlation between the two probes was very low (Pearson's R -0.05), suggesting that 5' and 3' sequences have high expression in different tumors.
**hsa-miR-30e-3p**	21	Incidence of tumor<br>16/21 target genes<br>p-value of overlap<br>3.3E-5	Cell movement<br>10/21 target genes<br>p-value of overlap<br>3.2E-4	1234<br>(593 positively<br>641 negatively)	Cell movement - predicted decreased<br>271 genes<br>activation z-score -2.3<br>p-value of overlap 3.8E-8	
